# Post-challenge insulin concentration is useful for differentiating between coronary artery disease and cardiac syndrome X in subjects without known diabetes mellitus

**DOI:** 10.1186/s13098-017-0209-1

**Published:** 2017-02-08

**Authors:** Kae-Woei Liang, Wayne H.-H. Sheu, Wen-Jane Lee, Wen-Lieng Lee, Hung-Chih Pan, I.-Te Lee, Jun-Sing Wang

**Affiliations:** 1 Cardiovascular Center, Taichung Veterans General Hospital, 1650 Taiwan Boulevard, Sec. 4, Taichung, 40705 Taiwan; 2 School of Medicine, National Yang Ming University, Taipei, Taiwan; 3 Department of Medicine, China Medical University, Taichung, Taiwan; 4 Division of Endocrinology and Metabolism, Department of Medicine, Taichung Veterans General Hospital, Taichung, Taiwan; 5 Institute of Biomedical Sciences, National Chung Hsing University, Taichung, Taiwan; 6 School of Medicine, National Defense Medical Center, Taipei, Taiwan; 7 Department of Medical Research, Taichung Veterans General Hospital, Taichung, Taiwan; 8 Tung-Hai University, Taichung, Taiwan; 9 School of Medicine, Chung-Shan Medical University, Taichung, Taiwan

**Keywords:** Coronary artery disease (CAD), Cardiac syndrome X (CSX), Insulin resistance (IR), Metabolic health, Oral glucose tolerance test (OGTT)

## Abstract

**Background:**

Cardiac syndrome X (CSX) is characterized by angina pectoris but with patent coronary arteries. Our previous study demonstrated that subjects with CSX had a higher fasting insulin-resistance (IR) than the controls. However, few studies have investigated the degree of IR, including oral glucose tolerance test (OGTT)-derived indices and profiles of metabolic abnormalities between CSX and coronary artery disease (CAD).

**Methods:**

Ninety-two CSX and 145 CAD subjects without known diabetes mellitus (DM) underwent coronary angiogram (CAG) for angina pectoris and also agreed to receive OGTT and glycated hemoglobin (HbA_1C_) evaluations for screening abnormal glucose regulation and IR.

**Results:**

CAD group had more subjects with metabolically unhealthy obesity (52.4 vs. 31.5%, p < 0.001) than the CSX group. The CAD group had higher OGTT 2 h glucose and insulin (both p < 0.005) while fasting glucose, insulin and HOMA-IR were similar to those of CSX subjects. In the binary regression analysis, OGTT 2 h insulin and being metabolic unhealthy were significantly different between the CAD and CSX groups, but there were no significant differences in Matsuda index, fasting glucose, insulin, HOMA-IR, or HbA_1C_.

**Conclusions:**

Post challenge OGTT 2 h insulin and being metabolic unhealthy were useful parameters in differentiating between CAD and CSX in subjects without known DM but suffered from angina pectoris and underwent CAG. Different degrees of IR and metabolic abnormalities might be implicated in the pathogenesis of micro vs. macro vascular coronary diseases.

*Trial registration* NCT01198730 at https://clinicaltrials.gov, Registered Sep. 8, 2010

## Background

Subjects with cardiac syndrome X (CSX) have angina-like symptoms with evidence of ischemia on stress electrocardiogram or isotope perfusion scan but with patent epicardial coronary arteries on coronary angiogram (CAG) [1–3]. The proposed mechanisms underlying CSX include endothelial dysfunction with impaired vaso-dilatory reserve in micro-vascular beds, inflammation, insulin resistance (IR), estrogen deficiency, or oxidative stress [4–6].

Studies have reported hyper-insulinemia during oral or intravenous glucose tolerance test was more prominent in the CSX group than in controls, which implies that IR might contribute to micro-vascular angina [7, 8]. Using hyper-insulinemia and the euglycemia clamp test, other investigators found that subjects with CSX had a higher degree of IR as compared to controls [9, 10]. In addition, CSX subjects more commonly had metabolic syndrome and related adiposity, metabolic, and inflammatory derangements [3, 11]. Our previous studies showed that CSX subjects had a higher IR, had more with hypertension, and a non-significantly higher body mass index (BMI) compared with the control group [6, 12]. Metabolic abnormalities and obesity increase the risk of future coronary artery disease (CAD) [13, 14]. Studies on non-diabetic participants showed that subjects with angiographically documented CAD exhibited moderate-severe IR and hyperinsulinemia [15, 16]. A study by Chauhan et al. [17] reported that there was a greater rise in insulin levels in response to a glucose challenge in those with CSX than in controls, and further showed that this rise was of a comparable magnitude to that found in subjects with obstructive CAD.

To date, few studies have investigated and compared the profiles of metabolic abnormalities and obesity or the differences in IR between CAD and CSX. Moreover, the usefulness of OGTT based IR or insulin-sensitivity indices, or glycated hemoglobin (HbA_1C_) for differentiating between CSX and CAD, who did not have known diabetes mellitus (DM), remains unexplored. The aims of the present study were to compare IR, including OGTT-derived indices, and profiles of metabolic abnormalities and obesity in CSX and CAD.

## Methods

### Study population

**Fig. 1:**
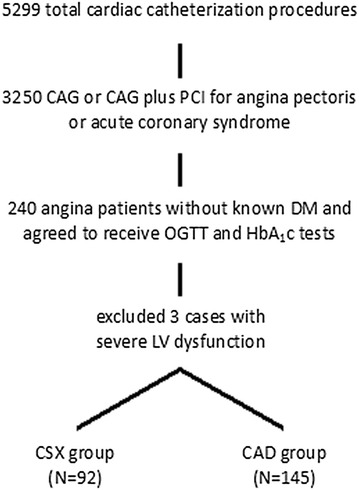
Flow chart of the study enrollment protocol. CAD: coronary artery disease; CAG: coronary angiogram; CSX: cardiac syndrome X DM: diabetes mellitus; HbA1C: glycated hemoglobin; LV: left ventricle; OGTT: oral glucose tolerance test; PCI: percutaneous coronary intervention

From April 2011 to May 2013, a total of 5299 cardiac catheterization procedures, including CAG, percutaneous coronary or peripheral vascular interventions, electrophysiological studies, catheter radiofrequency ablations, and pacemaker implantations, were performed at our catheterization laboratories. Among them, 3250 catheterizations were for CAG or CAG plus percutaneous coronary intervention for angina pectoris or acute coronary syndromes. Among the patients undergoing these procedures, 240 angina patients without known DM, who were admitted for CAG, agreed to receive OGTT and HbA_1C_ tests 2 weeks after hospital discharge (Fig. 1). Three of them were excluded in this analysis because of severe left ventricular dysfunction with ejection fraction lower than 35% and decompensated heart failure. Subjects with significant coronary stenosis (SYNTAX score >0) [18] or past histories of surgical or percutaneous coronary revascularization before the index admission were assigned to the CAD group (N = 145). Subjects with non-invasive tests for myocardial ischemia but with normal or near normal coronary angiograms (SYNTAX score = 0) [18] were assigned to the CSX (N = 92) (Fig. 1). We retrospectively reviewed all patients’ angiographic images, catheterization reports, and medical chart records. The study protocol was approved by the Human Research Review Committee of Taichung Veterans General Hospital (Taichung, Taiwan).

### Definition of metabolic health and obesity

The definition of obesity was BMI equal to or greater than 25 kg/m^2^ [19]. The evaluation of the number of metabolic abnormalities, including hypertension, high glucose, triglycerides, and low HDL-C, was done in accordance with the ATP III criteria [20]. Participants who met ≥2 of the following four criteria were considered metabolically unhealthy: high triglycerides (≥150 mg/dl) or taking lipid-lowering drugs, elevated systolic blood pressure (≥130 mmHg) or diastolic blood pressure (≥85 mmHg) or taking anti-hypertensive drugs, high fasting glucose (≥100 mg/dl) or taking medications for diabetes (insulin or oral anti-diabetic), and low HDL-C (<40 mg/dl for men and <50 mg/dl for women). The waist circumference criterion was not used because of its collinearity with BMI. We used this definition alongside data on BMI to create four phenotypes: metabolically healthy non-obesity (MHNO); metabolically healthy obesity (MHO); metabolically unhealthy non-obesity (MUHNO); metabolically unhealthy obesity (MUHO) [13, 21].

### Oral glucose tolerance test, glycated hemoglobin (HbA_1C_), insulin resistance indices, circulating adipokines, and high-sensitivity C-reactive protein (hs-CRP)

The study subjects underwent blood tests and OGTT after an overnight fast to investigate abnormal glucose regulation (AGR). After a fasting blood sample was collected, glucose load of 75 g was ingested over 5 min. Blood samples were collected at fasting, and 30 and 120 min after the test load. Blood glucose and insulin concentrations were measured in each sample. Serum insulin was determined by a commercially available assay kit (Roche Diagnostic, Mannheim, Germany). The detectable range was 0.2–1000 µIU/ml. The intra- and inter-assay coefficients of variation for insulin were 1.9–2.0% and 2.5–2.8%, respectively. Fasting insulin resistance was estimated using the homeostasis model assessment of IR (HOMA-IR), defined as fasting glucose mg/dl × fasting insulin μU/ml/405 [22, 23]. OGTT-derived Matsuda insulin-sensitivity index was defined as 10,000/sqrt [fasting glucose (mM) × fasting insulin (μU/ml) × mean glucose during OGTT (mM) × mean insulin during OGTT (μU/ml)] [24]. HbA1c was measured by boronate affinity high-performance liquid chromatography (CLC385TM, Primus Corporation, Kansas City, MO, USA). The intra- and inter-assay coefficients of variation for HbA1c [range 22 mmol (4.2%)–191 mmol/mol (19.6%)] were <0.9 and <2.9%, respectively. The diagnostic criteria for AGR were based on the American Diabetes Association definition [25]. Serum adiponectin, leptin and high-sensitivity C-reactive protein (hs-CRP) were determined by enzyme-linked immunosorbent assays (R&D Systems, Inc., Minneapolis, MN, USA). The intra- and inter-assay coefficients of variation for adiponectin were 3.53 and 6.50%, respectively, with a minimum detectable concentration of 0.079–0.891 ng/ml. The intra- and inter-assay coefficients of variation for leptin were 3.17 and 4.37%, respectively, with a minimum detectable concentration of <7.8 pg/ml. The intra- and inter-assay coefficients of variance for hs-CRP were 3.8–8.3% and 6.0–7.0%, respectively, with a mean minimum detectable concentration of 0.010 ng/ml.

### Statistical analysis

Continuous variables were expressed as median (interquartile range, 25th percentile to 75th percentile) for non-normally distributed data or as mean ± standard deviation for normally distributed data. Categorical data were expressed as percentages. Differences in non-normally distributed continuous variables between CAD and CSX were compared by Mann–Whitney U test while normally distributed data were analyzed by Student’s t test. Categorical variables were compared using the Chi square test or Fisher’s exact test as indicated. Binary logistic regression analyses were used to test significant variables associated with the diagnosis of CAD in comparison with CSX. Receiver operating characteristic curve (ROC) analysis was performed using OGTT 2 h insulin for differentiating the diagnosis of CAD vs. CSX. The SPSS (version, 12.1) statistical software package (SPSS, Inc., Chicago, IL, USA) was used for all calculations. A two-tailed p value of less than 0.05 was considered statistically significant.

## Results

### Baseline demographic data, inflammatory marker, and lipid profiles in subjects with CAD vs. CSX

The subjects in the CAD group were older and the proportions of male gender and hypertension were higher than those in the CSX group (Table 1). The CAD group had lower total cholesterol and low-density lipoprotein cholesterol (LDL-C), but more subjects were taking lipid-lowering medication before or after the index admission (p < 0.001) than those in the CSX group. Hs-CRP and the circulating adipokines were similar between the two groups (Table 1).

**Table 1:** Baseline demographic data of subjects with cardiac syndrome X vs. coronary artery disease (status without known diabetes mellitus)

	CSX (N = 92)	CAD (N = 145)	*p*
Age (years)	59 ± 11	62 ± 13	0.015
Gender (M/F)	62/30	132/13	<0.001
Hypertension (N) (%)	71 (77%)	134 (92%)	0.002
Systolic blood pressure (mmHg)	125 (114, 135)	128 (117, 141)	0.049
Body mass index (kg/m^2^)	25.0 (23.9, 27.3)	25.9 (23.7, 28.2)	0.174
Current smoking (N) (%)	51 (55%)	86 (60%)	0.563
Total cholesterol (mg/dl)	185 (165, 209)	165 (143, 193)	<0.001
LDL-C (mg/dl)	107 (91, 122)	90 (74, 112)	<0.001
HDL-C (mg/dl)	48 (43, 58)	43 (38, 51)	<0.001
Triglycerides (mg/dl)	122 (88, 173)	118 (88, 164)	0.960
Adiponectin (μg/ml)	3.7 (1.5, 6.0)	3.6 (1.5, 5.8)	0.583
Leptin (ng/ml)	4.2 (2.3, 8.5)	4.5 (2.8, 8.3)	0.627
Hs-CRP (mg/dl)	0.09 (0.02, 0.25)	0.12 (0.05, 0.26)	0.104
*Medication before index admission*			
Use of statin (N) (%)	13 (14%)	56 (39%)	<0.001
Use of fibrates (N) (%)	2 (2%)	1 (0.7%)	0.689
Use of anti-hypertensive medication (N) (%)	71 (77%)	134 (92%)	0.002
Medication post index admission			
Use of statin (N) (%)	22 (24%)	100 (69%)	<0.001

Non-normally distributed data are expressed as *median (interquartile range, 25th percentile to 75th percentile)*

*CAD* coronary artery disease, *CSX* cardiac syndrome X, *HDL-C* high-density lipoprotein cholesterol, *Hs-CRP* high sensitivity C-reactive protein, *LDL-C* low-density lipoprotein cholesterol, *Statin* HMG-CoA reductase inhibitors

### Revised diagnosis for abnormal glucose regulation after OGTT and HbA_1C_ tests for subjects with CAD vs. CSX (status without known DM)

Combining OGTT and HbA_1C_ tests, the subjects in the CAD group had more newly diagnosed DM (31.0 vs. 9.8%) but less newly diagnosed pre-DM (60.0 vs. 72.8%, p < 0.001) than those in the CSX group (Table 2).

**Table 2:** Revised diagnosis for abnormal glucose regulation after OGTT and HbA_1C_ tests for subjects with cardiac syndrome X vs. coronary artery disease (status without known diabetes mellitus)

	CSX (N = 92)	CAD (N = 145)	*p*
Normal (N) (%) (normal fasting glucose, OGTT & HbA_1C_)	16 (17.4%)	13 (9.0%)	<0.001
Newly diagnosed diabetes mellitus (N)(%) (fasting glucose, OGTT or HbA_1C_)	9 (9.8%)	45 (31.0%)	
Pre-diabetes (N) (%)	67 (72.8%)	87 (60.0%)	
IFG (N)	8	9	
IGT (N)	23	34	
IFG + IGT (N)	11	16	
HbA_1C_ (5.7–6.4%) (N)	55	83	

*CAD* coronary artery disease, *CSX* cardiac syndrome X, *HbA*_*1C*_ glycated hemoglobin, *IFG* impaired fasting glucose, *IGT* impaired glucose tolerance, *OGTT* oral glucose tolerance test

### Insulin resistance and sensitivity indices in subjects with CAD vs. CSX (status without known DM)

The fasting indices, including fasting glucose, insulin or HOMA-IR, were similar between the CSX and CAD groups. OGTT 30 min glucose and insulin levels were also similar between the two groups. However, subjects in the CAD group had higher values of HbA_1C_, OGTT 2 h glucose, and 2 h insulin and a trend (p = 0.054) toward lower OGTT-derived Matsuda insulin-sensitivity index than those in the CSX group (Table 3).

**Table 3:** Insulin-resistance indices in subjects with cardiac syndrome X vs. coronary artery disease (status without known diabetes mellitus)

	CSX (N = 92)	CAD (N = 145)	*p*
HbA_1C_ (%)	5.8 (5.6, 6.1)	6.0 (5.7, 6.4)	0.002
Fasting glucose (mg/dl)	93 (87, 100)	94 (89, 102)	0.115
OGTT 30 min glucose (mg/dl)	163 (146, 184)	165 (149, 188)	0.204
OGTT 2 h glucose (mg/dl)	127 (106, 155)	143 (114, 186)	0.004
Fasting insulin (µIU/ml)	8.3 (5.1, 13.8)	9.4 (6.0, 15.7)	0.317
OGTT 30 min insulin (µIU/ml)	65.0 (38.9, 99.9)	61.4 (39.1, 94.7)	0.817
OGTT 2 h insulin (µIU/ml)	47.3 (34.6, 75.9)	68.3 (40.1, 116.7)	0.002
HOMA-IR (mg/dl × µIU/ml)	2.0 (1.2, 3.3)	2.2 (1.3, 3.8)	0.231
OGTT derived insulin-sensitivity index, Matsuda (mM × µIU/ml)^−1^	87.1 (53.7, 143.0)	68.9 (46.8, 117.7)	0.054

Non-normally distributed data are expressed as *median (interquartile range, 25th percentile to 75th percentile)*

*HbA*_*1C*_ glycated hemoglobin, *OGTT* oral glucose tolerance test

### Obesity and metabolic health status in subjects with CAD vs. CSX (status without known DM)

Both the CAD and CSX groups had a high prevalence of obesity (58.6 vs. 50%, p = 0.228), but the CAD group had more metabolically unhealthy subjects (81.4 vs. 51.1%, p < 0.0001) than the CSX group. Moreover, the CAD group had a greater proportion of subjects with metabolically unhealthy obesity (52.4 vs. 31.5%), and fewer subjects with metabolically healthy obesity (6.2 vs. 18.5%, p = 0.001) in comparison with those in the CSX group (Table 4).

**Table 4:** Metabolic health and obesity status in subjects with cardiac syndrome X vs. coronary artery disease (status without known diabetes mellitus)

	CSX (N = 92)	CAD (N = 145)	*p*
			<0.001
MHNO	28 (30.4%)	18 (12.4%)	
MHO	17 (18.5%)	9 (6.2%)	
MUHNO	18 (19.6%)	42 (29%)	
MUHO	29 (31.5%)	76 (52.4%)	

*Metabolically unhealthy:* who met ≥2 of the following four criteria: high triglycerides (≥150 mg/dl) or taking lipid-lowering drugs, elevated systolic blood pressure (≥130 mmHg) or diastolic blood pressure (≥85 mmHg) or taking anti-hypertensive drugs, high fasting glucose (≥100 mg/dl) or taking medications for diabetes (insulin or oral anti-diabetic), and low high-density lipoprotein cholesterol (<40 mg/dl for men and <50 mg/dl for women)

*Obesity* body mass index equal to or greater than 25 kg/m^2^

*MHNO* metabolically healthy non-obesity, *MHO* metabolically healthy obesity, *MUHNO* metabolically unhealthy non-obesity, *MUHO* metabolically unhealthy obesity

### Binary logistic regression analysis of independent metabolic or IR variables for differentiating the diagnosis of CAD vs. CSX (status without known DM)

In the binary regression analysis, OGTT 2 h insulin concentration was significantly different between the CAD and CSX groups while the Matsuda index, HbA_1C_, fasting glucose, fasting insulin, OGTT 2 h glucose (p = 0.139) or HOMA-IR (p = 0.554) were not significantly different (Table 5). Being metabolically unhealthy was also a significant parameter for differentiating between CAD and CSX (Table 5).

**Table 5:** Binary logistic regression analysis of independent metabolic variables for diagnostic differentiation between coronary artery disease and cardiac syndrome X (status without known diabetes mellitus)

Factors	p value	OR	95% CI	
			Lower limit	Upper limit
*Mode 1. Metabolic health and HOMA-IR index* ^a, b^				
Age (years)	0.010	1.036	1.009	1.064
Gender (male vs. female)	<0.001	4.867	2.184	10.849
Body mass index (kg/m^2^)	0.449	1.036	0.946	1.135
Metabolically unhealthy (unhealthy vs. healthy)	0.004	2.680	1.380	5.204
HOMA-IR (mg/dl × µIU/ml)	0.554	1.022	0.950	1.099
Pre-admission statin use (with vs. without)	0.005	2.901	1.388	6.061
*Mode 2. Metabolic health and OGTT 2* *h glucose* ^a, c^				
Age (years)	0.045	1.029	1.001	1.058
Gender (male vs. female)	<0.001	5.169	2.290	11.669
Body mass index (kg/m^2^)	0.336	1.043	0.958	1.135
Metabolically unhealthy (unhealthy vs. healthy)	0.014	2.368	1.194	4.696
OGTT 2 h glucose (mg/dl)	0.139	1.006	0.998	1.013
Pre-admission statin use (with vs. without)	0.004	3.037	1.436	6.423
*Mode 3. Metabolic health and OGTT 2* *h insulin* ^a, d^				
Age (years)	0.012	1.036	1.008	1.065
Gender (male vs. female)	<0.001	6.544	2.763	15.502
Body mass index (kg/m^2^)	0.798	1.012	0.924	1.109
Metabolically unhealthy (unhealthy vs. healthy)	0.013	2.364	1.200	4.658
OGTT 2 h insulin (µIU/ml)	0.004	1.010	1.003	1.017
Pre-admission statin use (with vs. without)	0.005	2.939	1.381	2.657

*Metabolically unhealthy:* who met ≥2 of the following four criteria: high triglycerides (≥150 mg/dl) or took lipid-lowering drugs, elevated systolic blood pressure (≥130 mmHg) or diastolic blood pressure (≥85 mmHg) or took anti-hypertensive drugs, high fasting glucose (≥100 mg/dl) or took medications for diabetes (insulin or oral anti-diabetic), and low high-density lipoprotein cholesterol (<40 mg/dl for men and <50 mg/dl for women)

*CI* confidence interval, *HOMA-IR* homeostasis model assessment (HOMA) index of insulin-resistance = (fasting glucose mg/dl x fasting insulin μU/ml)/405, *OGTT* oral glucose tolerance test, *OR* odds ratio, *pre-index statin use* use of HMG-CoA reductase inhibitor before index admission

^a^Dependent variable: coronary artery disease vs. cardiac syndrome X

^b^Independent variables: demographic data, body mass index, metabolically unhealthy, statin use, and HOMA-IR

^c^Independent variables: demographic data, body mass index, metabolically unhealthy, statin use, and OGTT 2 h glucose

^d^Independent variables: demographic data, body mass index, metabolically unhealthy, statin use, and OGTT 2 h insulin

### Receiver operating characteristic curve analysis using OGTT 2 h insulin for differentiating CAD vs. CSX (status without known DM)

ROC curve analysis was performed using OGTT 2 h insulin for differentiating the diagnosis of CAD vs. CSX, the area under the curve was 0.618 (95% confidence interval 0.546–0.690, p = 0.002). Using OGTT 2 h insulin 64.9 µIU/ml as the cut-off value, the sensitivity was 57% for the diagnosis of CAD, and the specificity was 69% for the diagnosis of CSX. Adopting OGTT 2 h insulin of 145.6 µIU/ml as the cut-off value, the sensitivity was 15% for the diagnosis of CAD, and the specificity was 95% for the diagnosis of CSX.

## Discussion

The main finding of our study was that higher OGTT 2 h insulin level and being metabolically unhealthy were useful indices for differentiating between CAD and CSX in subjects undergoing CAG for angina pectoris but without known DM history. However, the Matsuda index, HbA_1C_, fasting glucose, insulin, OGTT 2 h glucose and HOMA-IR were not significant variables for distinguishing between CAD and CSX.

Our previous studies showed a high prevalence of newly diagnosed AGR in subjects undergoing CAG but without known DM [26, 27]. In this study, we further investigated and compared the prevalence of newly diagnosed DM, pre-DM, including impaired fasting glucose, impaired glucose tolerance or both, and abnormal HbA_1C_ in CAD vs. CSX. Combining OGTT and HbA_1C_ tests revealed a very high prevalence of newly diagnosed AGR in both the CSX and CAD groups. A novel finding in this study was that there was a greater proportion of newly diagnosed DM in the CAD group, but a higher prevalence of newly diagnosed pre-DM was found in the CSX group (Table 2). This implies that IR is one of the important mechanisms underlying the pathogenesis of both CAD and CSX and the different degrees of IR might contribute to the phenotype of DM vs. pre-DM as well as obstructive vs. non-obstructive coronary diseases.

Whether there is a difference in the prevalence of obesity or metabolic abnormalities between CSX and CAD has rarely been investigated. Moreover, there are inconsistent data on whether metabolically healthy obesity increases DM or cardiovascular risk to the same extent as metabolically unhealthy obesity does [13, 21]. In this study, we found a high prevalence of obesity in both CAD and CSX. CAD subjects had a higher prevalence of metabolic unhealthiness as well as metabolically unhealthy obesity in comparison with the CSX subjects (Table 4). Being metabolically unhealthy was a significant parameter for differentiating CAD vs. CSX (Table 5). The different prevalence rates of metabolic dysfunction, along with the difference in IR imply that the different degrees of metabolic derangements may underlie the development of macro- or micro-vascular disease in coronary trees.

A novel finding of this study showed that OGTT 2 h insulin was a significant parameter in the differential diagnosis between CAD vs. CSX while fasting glucose, insulin, HOMA-IR and HbA_1C_ were not. One study reported that HOMA-IR but not the Matsuda index was a predictor for CAD in subjects with angina but without DM undergoing CAG [28]. Another study reported that the CAD group had a higher OGTT post-challenge insulin level as compared with that of the non-CAD group [29]. Several population-based studies have applied fasting insulin level or HOMA-IR as a marker of insulin resistance and have shown significant associations with cardiovascular events in non-diabetic individuals, independent of other risk factors [30, 31]. One small-scale study reported that CSX and CAD subjects had a higher OGTT 2 h insulin than that of the controls, and CAD and CSX subjects had similar insulin responses in OGTT [17]. Our study revealed that CAD subjects had higher OGTT 2 h insulin levels, but the fasting IR indices were similar to those seen in CSX subjects. In the IR status, compensatory hyper-insulinemia might work only via the mitogenic pathway and promote vascular smooth muscle growth and migration [32], which would impair vaso-relaxation and contribute to the pathogenesis of CSX and CAD. Our study result implied that the degree and the difference in IR might be implicated in the obstructive vs. non-obstructive, as well in the micro-vascular vs. macro-vascular coronary disease. In OGTT test, 2 h insulin reflects the response of beta cells and peripheral tissues after a glucose challenge and is a better and more sensitive reflection of post-stimulated IR than fasting glucose or insulin [33]. Our study showed that the difference in IR between CAD and CSX was subtle and required a more refined test to determine.

Some studies used a stricter definition for CSX, excluding subjects with DM [2]. In this study, we used OGTT and A1C tests for screening for AGR in subjects with angina pectoris undergoing CAG but without known or overt DM. If we excluded newly diagnosed DM cases and reanalyzed the data, OGTT 2 h insulin remained a useful and significant IR parameter for differentiating between CAD and CSX.

There were several limitations in this study. This was a cross-sectional study that was conducted in a single catheterization laboratory within a specific time frame so selection bias could not be avoided. Because the study subjects needed to agree with OGTT tests post discharge and should not have known DM, only 237 patients were enrolled. The smaller sample size would limit its impact and interpretation. We did not measure c-peptide and thus the difference in circulating insulin concentration could not be attributed to the difference in beta cell production in response to glucose load or the difference in insulin clearance from circulation. Whether the difference in OGTT-based IR had an impact on cardiovascular outcome in the CAD or CSX subjects was not investigated in this study. The mechanisms why difference in IR appeared to reflect the clinical phenotypes of coronary disease, obstructive vs. non-obstructive, warrant further in vitro study. Moreover, we did not include some newly investigated markers, such as carotid intima media thickness, neutrophil and lymphocyte (N/L) ratios, or erectile dysfunction scores as comparing variables in this study [34, 35].

In conclusion, OGTT 2 h insulin level and metabolic unhealthiness were found to be useful diagnostic parameters for differentiating between CAD and CSX in subjects with angina pectoris undergoing CAG but without known DM, whereas fasting IR or HbA_1C_ indices were not. There were greater prevalence rates of metabolically unhealthy obesity and newly diagnosed DM among subjects with CAD compared with those of subjects in the CSX group.
